# Multitarget Therapeutics for Neurodegenerative Diseases

**DOI:** 10.1155/2020/6532827

**Published:** 2020-01-19

**Authors:** Moustafa T. Gabr, Samir Yahiaoui

**Affiliations:** ^1^Department of Radiology, Stanford University School of Medicine, Stanford, CA 94305, USA; ^2^Department of Drug Design and Optimization, Helmholtz Institute for Pharmaceutical Research Saarland, Campus E8.1, 66123 Saarbrücken, Germany

Neurodegenerative diseases such as Alzheimer's (AD), Huntington's (HD), and Parkinson's diseases (PD) are a group of progressive disorders that feature degeneration of the structure and function of the human nervous system. Impaired mitochondrial function, excessive oxidative stress in human brain, genetic factors, and malfunction in human brain metabolism contribute to the progression of neurodegenerative diseases [1]. Multitarget therapeutics hold promise in tackling the multifactorial and complex nature of neurodegenerative diseases [2–4]. The use of multitarget directed ligands (MTDLs) emerged in the recent years as a powerful strategy in the development of potential therapeutics for neurological disorders. A major advantage of MTDLs is their ability to act on multiple targets involved in the progression of these diseases in comparison to single target concept. For example, MTDLs have been successfully designed to reduce aggregation of both amyloid peptides and tau proteins which are the major pathological cascades proposed for neurological disorders. This special issue features comprehensive knowledge on recent research efforts in identifying multitargeted therapeutic approaches for neurodegenerative diseases.

We received articles from across the globe featuring interesting research in this area. A case-control study in this special issue demonstrated the potential role of peripheral immune disorders in the pathological progression of late-onset Parkinson's disease (LOPD). Moreover, development of phytomedicines as potential therapeutics for AD is discussed as well. The role of gut microbiota in progression of neurological disorders and approaches for regulation of this effect is featured. In addition, the guest editorial team contributed a detailed review on rational design of MTDLs for neurodegenerative diseases with special focus on Alzheimer's disease. This review explored a large number of promising MTDLs obtained based on different target combinations strategies.

In conclusion, there is a growing interest from researchers from different disciplines to identify efficient multitargeted strategies for neurodegenerative diseases. Comprehension of ongoing efforts in development of multitargeted strategies would enable scientists to identify the most successful approaches in the field and eventually lead to discovery of efficient therapeutics. The multidisciplinary nature of research in this area is evident in this special issue as it features research from various disciplines. The guest editorial team hopes that this special issue will help in featuring the multidisciplinary aspect of this research area and encourage future collaborative efforts.
